# Whey Protein, L-Leucine and Vitamin D Supplementation for Preserving Lean Mass during a Low-Calorie Diet in Sarcopenic Obese Women

**DOI:** 10.3390/nu14091884

**Published:** 2022-04-29

**Authors:** Elisabetta Camajani, Agnese Persichetti, Mikiko Watanabe, Savina Contini, Michaela Vari, Settimia Di Bernardo, Maria Faro, Carla Lubrano, Lucio Gnessi, Massimiliano Caprio, Sabrina Basciani

**Affiliations:** 1 PhD Program in Endocrinological Sciences, University of Rome “La Sapienza”, 00161 Rome, Italy; elisabetta.camajani@uniroma1.it; 2 Department of Human Sciences and Promotion of the Quality of Life, San Raffaele Roma Open University, 00166 Rome, Italy; massimiliano.caprio@sanraffaele.it; 3 Department of Experimental Medicine, Section of Medical Pathophysiology, Food Science and Endocrinology, Sapienza University of Rome, 00161 Rome, Italy; agnese.persichetti@gmail.com (A.P.); mikiko.watanabe@uniroma1.it (M.W.); savi.86@hotmail.it (S.C.); mvari86@gmail.com (M.V.); settimia.dibernardo@gmail.com (S.D.B.); maria.faro19@gmail.com (M.F.); carla.lubrano@uniroma1.it (C.L.); lucio.gnessi@uniroma1.it (L.G.); 4 Service of Pharmacovigilance, IRCCS-Regina Elena National Cancer Institute, 00144 Rome, Italy; 5 Laboratory of Cardiovascular Endocrinology, IRCCS San Raffaele, Pisana, 00161 Rome, Italy

**Keywords:** carbohydrate restriction, sarcopenia, obesity

## Abstract

In sarcopenic obese subjects it is essential to reduce body weight and preserve lean mass, in order to avoid a worsening of muscle function. Several studies have shown that leucine supplementation can be useful to improve skeletal muscle mass in sarcopenic patients. The aim of our study was to evaluate the effectiveness of a short-term low-calorie diet (LCD) combined with supplementation with whey protein and leucine on weight loss, lean mass and muscle strength in sarcopenic, obese, hyperinsulinemic and post-menopausal women. Sixteen females with a mean age of 60 years (range: 50–70 years), BMI 37.6 kg/m^2^ (range: 31.7–44.1 Kg/m^2^), HOMA-index ≥ 2.5 (range: 2.9–12) were assigned to an LCD regimen (1000 kcal/day) with supplementation of 18 g whey proteins which 4.1 g of leucine for 45 days. Anthropometric indexes, blood and urine chemistry, body composition by DEXA, muscle strength by handgrip test and Short Physical Performance Battery (SPPB) were assessed at baseline and at the end of the treatment. A significant reduction in BMI (37.6 vs. 35.7 Kg/m^2^), waist circumference (107 vs. 102.4 cm), HOMA index (4.8 vs. 2.3) and fasting insulin (17.4 vs. 10.4 μIU/mL) was observed in all patients. Women preserved total lean body mass (55 vs. 5%) and significantly improved their muscle strength, as measured by handgrip (15.3 vs. 20.1 Kg), and their muscle function, as measured by SPPB (7.5 vs. 8.9). A significant increase in BUN was also observed (36.1 vs. 46.3). We conclude that LCD with adequate protein intake and supplementation with whey protein and leucine should be promoted to maintain muscle mass and improve muscle strength in post-menopausal women with sarcopenic obesity.

## 1. Introduction

The term “sarcopenia” comes from the Greek ‘σαρξ’ (meat) and ‘πενια’ (lost). This term was first proposed by Rosenberg in 1988 and originally indicated only the loss of muscle mass caused by aging [1]. In 2010, the European Working Group on Sarcopenia in Older People (EWGSOP) defined sarcopenia as a syndrome characterized by progressive and generalized loss of skeletal muscle mass and strength, with a risk of adverse outcomes such as physical disability, poor quality of life and death [2]. Muscle loss related to aging is the result of the reduction in the size and number of muscle fibers, probably determined by multiple factors dependent on physical inactivity, inadequate nutritional intake, oxidative stress, systemic inflammation and hormonal changes [3,4,5,6]. In the updated definition of sarcopenia in 2019, EWGSOP2 not only refers to sarcopenia as the loss of muscle mass but focuses more on the loss of muscle strength [7]. EWGSOP2 indicates low foreground strength as a primary marker of probable sarcopenia, and sarcopenia is now considered a veritable disease of skeletal muscle (muscle failure), with low muscle strength that overcomes the role of low muscle mass as the main determinant. Muscle mass, strength and physical performance represent the measurable readouts to define sarcopenia [2]. In recent years, the literature has been referring to Sarcopenic Obesity (SO) which is a clinical condition characterized by an excess of fat mass and a reduction in muscle mass [8,9,10,11]. As reported in European Consensus on Definition and Diagnosis of Sarcopenia, SO is most often reported in older people, since its prevalence increases with age. Obesity exacerbates sarcopenia, increases the infiltration of fat in skeletal muscle, lowers physical function and increases the risk of mortality [3]. Moreover, as reported by Stoklossa et al., sarcopenia can be masked by obesity: a reduction in muscle strength and muscle function could therefore occur without any evidence of a reduction in muscle mass [12]. Currently, there is no univocal definition of SO in terms of diagnostic criteria and cut-offs; for this reason, it is still not possible to determine its prevalence [13]. Therefore, if sarcopenia has long been associated with aging and older people, it is now recognized that its development begins earlier in life and that the sarcopenic phenotype has several contributing causes beyond aging. Muscle and strength loss in women after menopause is the result of multiple factors, mostly dependent on physical inactivity, malnutrition, mitochondrial stress, systemic inflammation and hormonal changes that can also contribute to obesity [14].

For a sarcopenic obese subject, a lifestyle modification with adequate nutrition and proper physical activity is essential to counteract its progression. According to the Italian and International Guidelines, the administration of 0.8–1.1 g of protein per kg of body weight, depending on the age group, is sufficient to avoid muscle catabolism and support muscle mass [15,16]. According to Batsis and Villareal, strategies that optimize protein anabolism during weight loss, such as spreading protein throughout the day, can prevent weight loss-induced sarcopenia [9]. In addition, the increase in dietary protein intake also stimulates muscle protein synthesis. As reported by Ganapathy and Nieves, several studies have found an association between sarcopenia and protein intake, with lower protein intake associated with a loss of lean mass by DXA and a reduced grip strength [17].

The source of protein, the timing of intake, and specific amino-acid constitution also represent critical factors in increasing muscle mass and strength [5]. Recent studies have shown how protein supplementation, especially with high leucine content, can be effective in degenerative and end-stage diseases. Cancer cachexia induces a variety of metabolic disorders, including skeletal muscle imbalance. In this view, nutritional supplementation with leucine shows a modulatory effect over tumor-induced derangements in vivo and in vitro [18]. In fact, leucine directly affects skeletal muscle anabolism through activation of the mechanistic target of rapamycin complex 1 (mTORC1) signaling [19,20,21,22]. Xu et al. confirmed the efficacy of leucine on muscle protein synthesis, lean body mass and leg lean mass in older people [23].

On the basis of these considerations, we hypothesized that a low-calorie diet (LCD) with whey protein, leucine and Vitamin D supplementation is strictly linked to the improvement of sarcopenic obesity in post-menopausal women. The primary outcome was represented by the preservation of lean mass and muscle strength.

## 2. Materials and Methods

### 2.1. Study Design

This was an open, nutritional intervention, uncontrolled, pilot study that enrolled sarcopenic, obese, hyperinsulinemic and post-menopausal women among those attending the Center for the Study of Eating Disorders and Obesity, Department of Experimental Medicine, Section of Medical Pathophysiology, Food Science and Endocrinology of the University of Rome “La Sapienza,” Italy. This trial was registered at clinicaltrials.gov, accessed on 26 March 2022 (NCT05287659).

### 2.2. Inclusion Criteria

The inclusion criteria were as follows: women, age between 50 and 70 years, BMI between 30 and 40 kg/m^2^ with stable body weight (BW) in the previous 6 months, with hyperinsulinism (HOMA-IR ≥ 2.5) and sarcopenia. The presence of SO was considered when the following conditions were satisfied:

- Fat mass > 38%, according to NHANES III [24]
- Handgrip < 16 Kg, according to EWGSOP2 [7]
- Chair stand test > 15 s, according to EWGSOP2 [7]
- SPPB < 8, according to EWGSOP2 [7]

### 2.3. Patients

We screened 350 subjects for eligibility; 334 were excluded (298 did not meet all the inclusion criteria and 36 declined to participate). Only 16 subjects were enrolled for this study. All patients had a very low level of physical activity and were sedentary. During this study, patients did not follow any scheduled physical activity.

### 2.4. Anthropometric Assessment

BW, height, systolic and diastolic BP, waist circumference (WC), thigh circumference (TC), and hip circumference (HC) were measured at T0 and every 2 weeks for 45 days. Anthropometric measurements were recorded after an overnight fast under resting conditions using calibrated equipment. BW was measured using a balance-beam scale (Seca GmbH & Co, Hamburg, Germany) [25]. Systolic and diastolic BP were measured using a mercury-gravity manometer. The height was rounded to the closest 0.5 cm. BMI was calculated as weight divided by squared height in meters (kg/m^2^). WC was measured midway between the costal arch and the iliac crest, HC was measured at the symphysis-greater trochanter level to the closest 1 cm, and TC was measured at the middle of the right thigh.

### 2.5. Blood and Urine Chemistry

Blood count (ADVIA 2120i Hematology System, Siemens Healthcare s.r.l., Milano, Italy), electrolytes (chloride, potassium, and sodium: indirect ion-selective electrode potentiometry; calcium, and magnesium: colorimetric assay), glucose (enzymatic colorimetric assay), insulin (electrochemiluminescence immunoassay), lipids (triglycerides, total, high-density lipoprotein, and low-density lipoprotein cholesterol; enzymatic colorimetric assay), total protein and albumin (capillary system), C-reactive protein (immunoturbidimetric assay), erythrocyte sedimentation rate (capillary photometric assay), plasma creatinine (kinetic colorimetric compensated Jaffé method), blood urea nitrogen (BUN), uric acid, alanine transferase, and aspartate transaminase (enzymatic colorimetric assay), and estimated glomerular filtration rate (eGFR) were determined at baseline and T45 [26]. All analyses were performed on a COBAS 6000 (Roche Diagnostics, Risch-Rotkreuz, Switzerland) and on CapillarysR Systems (Sebia, Evry, France). Insulin resistance was determined using HOMA-IR [27].

### 2.6. Dual-Energy X-Ray Absorptiometry Measurement

Body composition, total and regional body fat mass and fat-free mass were measured by dual-energy X-ray absorptiometry (Hologic 4500, Bedford, MA, USA) at baseline and at the end of the trial. Trunk fat was defined as the adipose tissue localized within the region below the chin, delineated by vertical lines within the left and right glenoid fossae bordering laterally to the ribs and by the oblique lines that cross the femoral necks and converge below the pubic symphysis.

### 2.7. Muscular Strenght and Functional Tests

Handgrip strength (HG) was measured with a digital dynamometer (DynEx, Akern, Pontassieve, FI, Italy) at T0 and T45 with the patients seated, shoulder adducted, and forearms resting flat on the chair arms. Before starting, patients were asked to squeeze the dynamometer as hard as possible for at least 3 s. Three measurements were repeated with both the dominant and nondominant arms. The highest value measured for the dominant arm was recorded; the handgrip strength test was considered impaired when it was <16 kg [7].

The SPPB battery consists of three components of physical performance: (1) standing balance, (2) chair stands, and (3) gait speed. A score from 0 (poorest) to 4 (best) was assigned for each of these three components. The sum of the scores provided a composite score ranging from 0 to 12; physical performance was considered impaired when the total SPPB score was ≤8 [1,2].

### 2.8. Dietary Intervention

All patients followed an LCD dietary regimen (1000 kcal/day).

Given the inadequate leucine content normally present in food (such as meat, fish and eggs) for this high-risk population, such as postmenopausal obese sarcopenic women, a protein supplement containing 18 g of whey protein was given, including 4 g of leucine, to satisfy the correct protein intake. In this way, it was possible to give 1.38 g protein/kg body weight/day.

Hence, the dietary macronutrient composition was based on 28% protein (70 g/day), 32% fat (33.3 g/die) and 30% carbohydrate (97.7 g/die) for 45 days.

The powdered drink preparation was characterized by whey protein (18 g), leucine (4.1 g) and vitamin D3 (5.01 mcg, corresponding to 200 IU), which was taken at 5 p.m. Therefore, the total amount of vitamin D provided to patients through diet and supplementation reached a total of 600 IU/day (as required by LARN 2014) [15].

It was recommended to drink no less than 1.5–2.0 l of water per day.

Since in the program fruit consumption was limited, the patients were instructed to assume a multi-vitamin and multi-saline supplement (containing 365 mg magnesium) formulated to maintain the physiological acid/base balance and it was taken in the morning.

### 2.9. Data Management and Statistical Methods

Data are expressed as the mean values ± standard deviations or percentages where appropriate. Comparisons between groups were evaluated using the Student’s *t*-test. The number of subjects was identified considering the number of subjects generally included in similar published pilot studies. Differences were considered statistically significant when *p* was ≤0.05.

### 2.10. Ethical Aspects

The study protocol was approved by the Ethics Committee of the University of Rome “La Sapienza” (code 3920) and was conducted in accordance with the Declaration of Helsinki and Good Clinical Practice. All patients were informed about the possible risks and benefits of the proposed interventions. All patients signed an informed consent form in accordance with the General Data Protection Regulation (GPDR, 2016/679).

## 3. Results

Sixteen females with a mean age of 60 years (range: 50–70 years) were enrolled in the study. All patients were post-menopausal, sarcopenic, insulin-resistant (HOMA index was 4.8 ± 2.2) and with medium degree obesity (average BMI was 37.6 ± 4.4 kg/m^2^). Three patients (18.7%) were dyslipidemic and were treated with statins and eight (37.5%) were on antihypertensive therapy, no one was diabetic.

The differences in anthropometric measures, weight, blood pressure, clinical chemistry and blood count at the beginning and at the end of the study are shown in Table 1.

At the end of the observation period, body weight was improved with a significant change in waist circumference and BMI (*p* = 0.04 and *p* = 0.042, respectively). The average weight loss was 4.6% (range 9.2–1.4%) in 45 days.

Fasting insulin and HOMA IR were significantly improved at the end of the study (*p* = 0.001 and *p* = 0.001, respectively). Creatinine and eGFR did not undergo any significant change, conversely, BUN was increased (*p* = 0. 026). Lipid profile (total cholesterol, LDL cholesterol, triglycerides) and inflammatory markers were improved at the end of the study. Electrolytes did not vary significantly, except for Na and Mg which showed a slight reduction and increase (*p* = 0.030 and *p* = 0.002, respectively), however, all remained in the normal range.

Body composition parameters underwent an overall improvement, with a significant decrease in total trunk fat (*p* = 0.049), as shows in Table 2.

**Figure 1:**
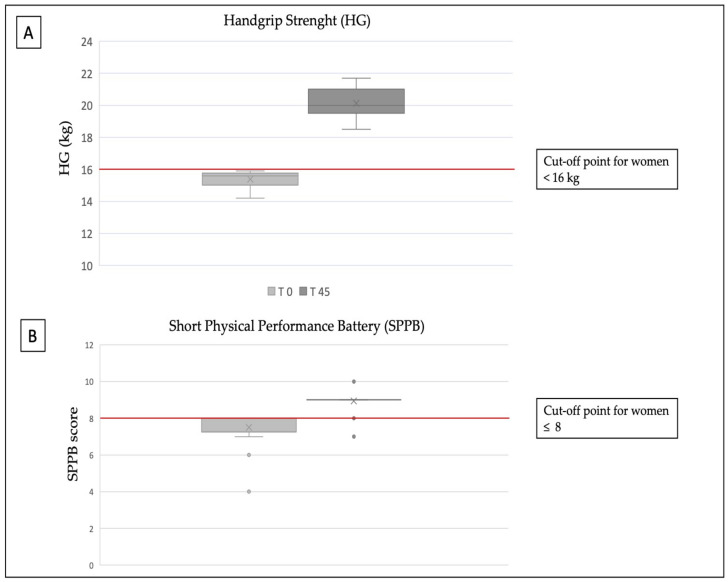
Functional test: (**A**) Handgrip; (**B**) Short Physical Performance Battery (SPPB).

Figure 1 shows the results of the functional tests. Muscle strength measured by dynamometry improved significantly (average handgrip test at baseline was 15.3 ± 0.5 kg; average handgrip test after 45 days was 20.1 ± 0.9 kg; *p* = 0.000) from the baseline. The score of the SPPB test was significantly improved (range of total SBBP at T0 was 4–9; the range of total SPPB at T45 was 8–10; *p* = 0.000) after 45 days.

None of the patients dropped out of the study and no significant adverse events were recorded.

## 4. Discussion

Low physical activity, low protein intake and elevated oxidative stress, in association with a decline in estrogen, represent the greatest contributors to sarcopenia in postmenopausal women [28,29,30,31]. In addition, the simultaneous presence of both low muscle mass and obesity confers a higher risk of functional impairment and disability in this phase of a woman’s life. Few studies in the literature report nutritional interventions or physical rehabilitation for obese adults with sarcopenia [32,33,34,35]. According to National and International guidelines, the Recommended Daily Allowance (RDA) for daily dietary protein for adults is 0.8–1.1 g/kg of reference body weight/day; however, at present, there are many doubts that this regimen may be adequate for elderly subjects or for those with sarcopenia [36,37]. Although the RDA protein is sufficient to maintain weight, a low-protein and low-calorie diet can cause weight loss and muscle mass loss, especially if repeated. In the Health ABC study cohort, among participants who lost weight during the three-year follow-up period, a higher protein intake was associated with lean mass loss. Finally, among individuals who gained weight during the 3-year follow-up, an increase in protein intake was associated with higher lean body mass [38]. Moreover, as reported in the review by Trouwborst and colleagues, it is well established that the intake of dietary amino acids, and especially the essential amino acids, has a positive regulatory effect on the muscle protein synthesis in the muscle [33]. As reported by Wall and colleagues, whey protein has been shown to be very effective in stimulating postprandial muscle protein accretion in older men [39]. In addition, an intake of about 2.0–2.5 g/day leucine, mainly derived from animal sources, improves the post-prandial muscle protein synthesis in elderly men. Moreover, a recent review by Cereda and colleagues demonstrated that oral supplementation with whey protein, leucine and vitamin D is an effective therapy for older patients with sarcopenia and should be offered as a first-line treatment, not only to improve clinical outcomes but also to reduce healthcare resource consumption [40]. In our research, a high-protein diet (1.4 g/kg reference body weight/day) containing a supplementation with 18 g of high biological value whey protein and 4.1 g of leucine improved body composition by reducing absolute fat mass and preserving lean mass. Patients on a high protein diet showed a significant increase in muscle strength and function after 45 days of treatment. In addition, our data show that a 45-day-long LCD supplemented with whey protein rich in leucine causes a significant reduction in BMI and improves glycemic control in patients with obesity and insulin resistance.

The LCD was safe and well-tolerated for 45 days; however, a significant increase in BUN was found, and a small increase in serum creatine and a mild reduction in eGFR were found, although not statistically significant.

Therefore, this nutritional approach requires strict surveillance until definitively proven safe. Moreover, it could be advisable to vary the animal protein sources and increase the use of vegetable proteins [41].

Leucine supplementation substantially contributes to preserving muscle function and performance [39]. In line with the existing literature, we cannot exclude the fact that dietary magnesium may also support the preservation of skeletal muscle mass and the improvement of muscle function [17]. As reported by Welch et al., dietary magnesium is important for skeletal muscle function due to its direct role in muscle metabolism and indirect interaction with chronic low-grade inflammation, which represents a risk factor for loss of mass, skeletal muscle strength and function [42]. In line with this, Peterman-Rocha et al. revealed an inverse association between magnesium intake and sarcopenia [43].

It is evident that a correct diet supported by adequate supplementation of micro- and macro-nutrients seems to be relevant for the treatment of sarcopenic obese subjects. As remarked by Ganapathy et al., numerous studies evaluated the impact of supplementation with a combination of several macro- and micro-nutrients (whey protein, leucine, vitamin D, vitamin C, B-vitamin complex, calcium, magnesium, Omega 3 Fatty Acid) with regard to muscle mass and strength [17].

The aim of this pilot study was to identify an ideal macro- and micro-nutrient pattern that could be helpful for a high-risk population, such as postmenopausal obese sarcopenic women. It is also true that the primary treatment for sarcopenia is represented by physical exercise, which has been shown to produce the most beneficial preventive and therapeutic effects [44,45]. The contribution of both physical activity and diet are necessary for maintaining muscle function and endocrine function [46,47]. As regards physical exercise, not a single type of exercise appears to adequately address the requirements of therapeutic exercise in age-related sarcopenia, and therefore well-rounded exercise programs, consisting of aerobic and resistance exercises should be preferred. A limitation of this study is that it did not include daily physical activity for the patients.

However, the study has numerous limitations represented by the number of patients, the duration of the study and the lack of follow-ups. Another limitation is the absence of a control arm, and for this reason, the current results must be considered with caution. In the future, longitudinal and randomized case-control studies will be performed to confirm the results obtained in this first exploratory study.

## 5. Conclusions

We conclude that an LCD with adequate protein intake and a concomitant supplementation with whey protein, leucine and optimal micronutrient combination, such as Vitamin D, displays favorable effects in post-menopausal women with sarcopenic obesity, preserving muscle mass and improving muscle strength and function.

## Figures and Tables

**Table 1:** Participant characteristics of anthropometric measurements and blood test at baseline (T0) and at the end of the study (T45).

	T 0	T 45	*p* < 0.05
Weight (Kg)	96.8 ± 12.9	91.3 ± 13.1	0.162
Body Mass Index (Kg/m^2^)	37.6 ± 4.4	35.7 ± 4.2	0.044 *
Waist Circumference (cm)	107 ± 7.4	102.4 ± 7.3	0.042 *
Thigh Circumference (cm)	62.4 ± 2.8	60.4 ± 2.6	0.046 *
Systolic Body Pressure (mmHg)	127 ± 11	120 ± 11	0.066
Diastolic Body Pressure (mmHg)	75 ± 7	70 ± 10	0.060
Fasting Glycemia (mg/dL)	112 ± 23.9	105.4 ± 14.2	0.145
Fasting Insulin (μIU/mL)	17.4 ± 7.7	10.4 ± 3.9	0.001 *
HOMA Index	4.8 ± 2.2	2.3 ± 1.1	0.001 *
Hb1AC (%)	5.96 ± 0.6	5.67 ± 0.3	0.053
BUN (mg/dL)	36.1 ± 7.8	46.3 ± 18.5	0.026 *
Creatinine (mg/dL)	0.79 ± 0.2	0.86 ± 0.2	0.192
eGFR	124.2 ± 36.2	110.2 ± 37.2	0.140
Na (mmol/L)	142.1 ± 2.1	140.7 ± 2	0.030 *
K (mmol/L)	4.4 ± 0.3	4.5 ± 0.3	0.280
Cl (mmol/L)	100 ± 1.7	99 ± 1.8	0.076
Ca (mg/dL)	9.6 ± 0.4	9.7 ± 0.5	0.230
Mg (mg/dL)	2.0 ± 0.1	2.2 ± 0.1	0.002 *
P (mg/dL)	3.5 ± 0.5	3.7 ± 0.4	0.100
AST (U/L)	17.5 ± 5.4	19.5 ± 4.2	0.128
ALT (U/L)	21.2 ± 12.6	23.2 ± 11.4	0.321
Total Cholesterol (mg/dL)	224 ± 56	207 ± 47	0.177
LDL Cholesterol (mg/dL)	140 ± 53	128 ± 47	0.243
HDL Cholesterol (mg/dL)	57 ± 13	55 ± 11	0.294
Triglycerides (mg/dL)	132 ± 39	120 ± 35	0.192
Uric acid (mg/dL)	5.2 ± 1.4	5.3 ± 1.4	0.424
CRP (mcg/L)	7686 ± 6509	5643 ± 4973	0.166
ESR (mm/h)	36 ± 15	38 ± 18	0.412

Abbreviations: BUN, blood urea nitrogen; eGFR, estimated Glomerular Filtration Rate; AST, aspartate transaminase; ALT, alanine transferase; CRP, C reactive protein; ESR, erythrocite sedimentation rate. All values are presented as mean ± standard deviation. * *p* < 0.05.

**Table 2:** Body composition by DXA at T0 and T45.

	T 0	T 45	*p* < 0.05
Left Arm Fat (%)	53 ± 5.2	49 ± 5.5	0.022 *
Left Arm Lean (%)	43 ± 7.1	48 ± 4.9	0.013 *
Left Arm Fat (g)	3040 ± 806	2733 ± 805	0.145
Left Arm Lean (g)	2015 ± 411	2199 ± 324	0.085
Right Arm Fat (%)	49 ± 5.3	47 ± 5.5	0.115
Right Arm Lean (%)	46 ± 4.7	49 ± 5.2	0.060
Right Arm Fat (g)	2905 ± 763	2618 ± 726	0.142
Right Arm Lean (g)	2197 ± 320	2166 ± 307	0.192
Left Leg Fat (%)	44 ± 4.4	41 ± 4.5	0.077
Left Leg Lean (%)	49 ± 13	56 ± 6	0.029 *
Left Leg Fat (g)	6886 ± 1719	5727 ± 1863	0.038 *
Left Leg Lean (g)	6967 ± 1206	7361 ± 1262	0.186
Right Leg Fat (%)	43 ± 5	41 ± 4.7	0.196
Right Leg Lean (%)	54 ± 4.9	56 ± 4.5	0.161
Right Leg Fat (g)	6812 ± 1754	6291 ± 1702	0.200
Right Leg Lean (g)	7488 ± 1243	7417 ± 1305	0.437
Trunk Fat (g)	21064 ± 4187	18517 ± 4266	0.049 *
Trunk lean (g)	27872 ± 2871	27682 ± 3309	0.431
Trunk Fat (%)	42 ± 3.9	39 ± 4.5	0.026 *
Trunk lean (%)	56 ± 3.8	59 ± 4.4	0.026 *
Total Fat (g)	41797 ± 7705	37513 ± 7706	0.063
Total Lean (g)	53182 ± 5496	53020 ± 6088	0.468
Total Fat (%)	42 ± 3.3	40 ± 3.2	0.016 *
Total Lean (%)	55 ± 3.2	57 ± 3.1	0.017 *

* *p* < 0.05.

## Data Availability

Not applicable.
